# Regulation of the DLC3 tumor suppressor by a novel phosphoswitch

**DOI:** 10.1016/j.isci.2024.110203

**Published:** 2024-06-06

**Authors:** Yannick Frey, Cristiana Lungu, Florian Meyer, Franziskus Hauth, Daniel Hahn, Corinna Kersten, Vivien Heller, Mirita Franz-Wachtel, Boris Macek, Igor Barsukov, Monilola A. Olayioye

**Affiliations:** 1 University of Stuttgart, Institute of Cell Biology and Immunology, Stuttgart, Germany; 2 University of Stuttgart, Stuttgart Research Center Systems Biology, Stuttgart, Germany; 3 University of Liverpool, Institute of Systems, Molecular and Integrative Biology, Department of Biochemistry, Cell and Systems Biology, Liverpool, UK; 4 Proteome Center Tübingen, University of Tübingen, Tübingen, Germany

**Keywords:** Molecular interaction, Cell biology, Proteomics

## Abstract

Deleted in liver cancer 3 (DLC3) is a Rho GTPase-activating protein (RhoGAP) that plays a crucial role in maintaining adherens junction integrity and coordinating polarized vesicle transport by modulating Rho activity at the plasma membrane and endomembranes. By employing bioinformatical sequence analysis, *in vitro* experiments, and *in cellulo* assays we here identified a polybasic region (PBR) in DLC3 that facilitates the association of the protein with cellular membranes. Within the PBR, we mapped two serines whose phosphorylation can alter the electrostatic character of the region. Consequently, phosphomimetic mutations of these sites impaired the membrane association of DLC3. Furthermore, we found a new PBR-dependent localization of DLC3 at the midbody region, where the protein locally controlled Rho activity. Here, the phosphorylation-dependent regulation of DLC3 appeared to be required for proper cytokinesis. Our work thus provides a novel mechanism for spatiotemporal termination of Rho signaling by the RhoGAP protein DLC3.

## Highlights

• A polybasic region (PBR) regulates the membrane binding of the RhoGAP DLC3
• Phosphorylation of DLC3’s PBR impairs its membrane association
• DLC3 localizes to the midbody region and controls Rho activity during cytokinesis
• Proper cytokinesis requires phosphorylation-dependent regulation of the DLC3 PBR

## Graphical abstract

**Figure:**
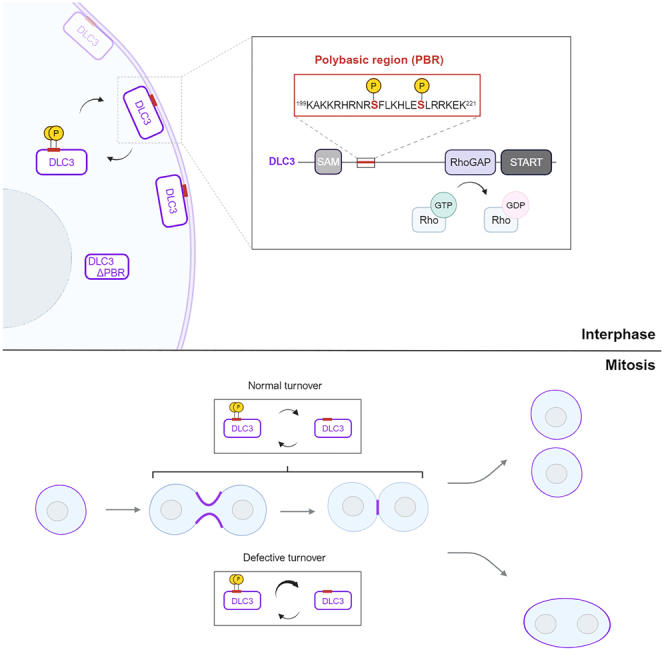

## Introduction

Members of the Rho subfamily of small GTPases, including RhoA, Rac1, and Cdc42, integrate a wide range of extracellular stimuli and translate these into local changes in cytoskeleton dynamics. Thereby, Rho GTPases directly and indirectly regulate most cellular processes involving actin and microtubule remodeling, such as cell morphology and motility, membrane trafficking, cell division, and gene transcription.^1^^,^^2^ Rho GTPases are activated by guanine nucleotide exchange factors (GEFs) that promote the exchange of GDP for GTP. This results in Rho association with membranes, binding of effector proteins and downstream pathway activation. Conversely, GTPase-activating proteins (GAPs) catalyze the hydrolysis of bound GTP, thus returning the GTPase to the inactive state and termination of downstream signaling.^3^ Although the general principles of Rho GTPase activity switching are well established, the specific molecular mechanisms that control Rho signaling dynamics and give rise to Rho activity gradients at cellular membranes are only partially understood.

In this context, the deleted in liver cancer (DLC) family of RhoGAP proteins has gained attention because of its tumor suppressive function, the three family members being frequently downregulated in several types of human cancers.^4^^,^^5^^,^^6^ Among the family members, DLC3 stands out due to its ability to localize to different cellular membranes, where it plays specific functions. For example, our previous work has revealed that local DLC3 activity at the plasma membrane is vital for the maintenance of adherens junctions and cell polarity and that the basolateral polarity protein Scribble is responsible for DLC3 recruitment to these sites.^7^^,^^8^ Furthermore, DLC3 was found to associate with endomembrane compartments through SNX27 adaptor protein binding, where the regulation of local Rho signaling controls endocytic recycling.^9^^,^^10^^,^^11^ The interaction with either Scribble or SNX27 is mediated via a C-terminal PDZ (PSD-95, Discs large, ZO-1) ligand motif.^8^^,^^10^ While DLC3 was reported to activate both RhoA and Cdc42 *in vitro*, studies using Förster resonance energy transfer (FRET) activity biosensors in cells detected activity toward RhoA and the closely related family member RhoB.^10^^,^^12^^,^^13^ Moreover, DLC3’s significance extends beyond cancer, with recent research describing its conserved role in male gonadogenesis.^14^^,^^15^

Although specific adaptor proteins directing DLC3 to distinct subcellular sites have been identified, the general molecular principles regulating DLC3 binding to and detachment from membranes and how this relates to Rho activity patterns are still not fully understood. Here, we identify in DLC3 an aminoterminal PBR facilitating its membrane binding in a phosphorylation-dependent manner. We furthermore find a novel localization of DLC3 at the midbody region, which is PBR dependent and plays a role in cell division. Together, our findings contribute to a better understanding of DLC3 biology and demonstrate how the timing of RhoGAP membrane association regulates Rho signaling and consequently cellular homeostasis.

## Results

### A novel polybasic region in DLC3 is important for membrane binding

**Figure 1:**
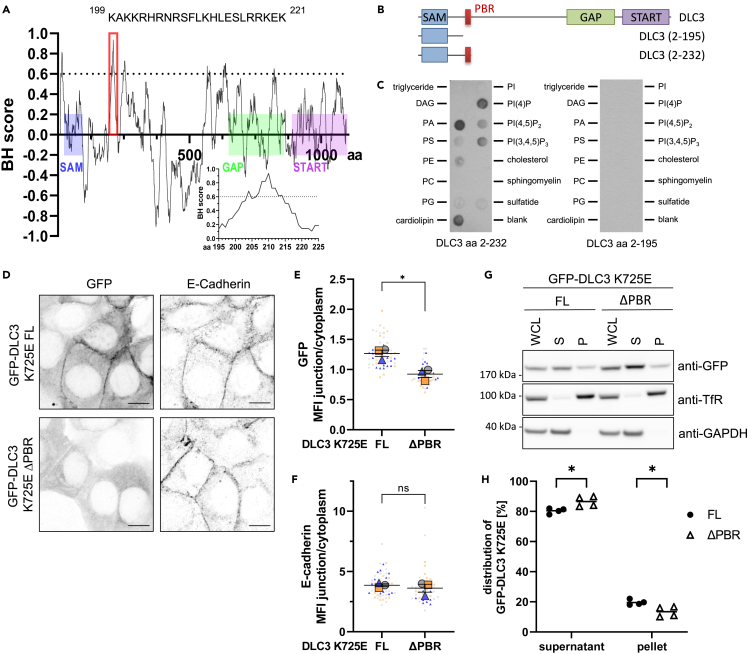
Regulation of DLC3 membrane association by a novel polybasic region (A) BH plot of basic and hydrophobic residues in DLC3 using the scale developed by Brzseska et al. The relative localization of the SAM, GAP and START domains are schematically annotated on the profile. The red box marks the identified polybasic region (PBR) spanning amino acids (aa) 199–221 with the sequence given. The blot of this region is magnified in the insert. (B) Line diagram showing the domain organization of full-length DLC3 and fragments used for the lipid overlay assay in (C), with the PBR marked in red. (C) Recombinant GST-tagged N-terminal DLC3 fragments containing the PBR (left) or lacking the PBR (right) were incubated with lipid strips. Bound protein was detected by immunoblotting with anti-GST antibody, followed by HRP-coupled secondary antibody. DAG = diacylglycerol, PA = phosphatidic acid, PS = phosphatidylserine, PE = phosphatidylethanolamine, PC = phosphatidylcholine, PG = phosphatidylglycerol, PI = phosphatidylinositol, sulfatide = 3-sulfogalactosylceramide. (D) Localization of GFP-DLC3 K725E full-length (FL) and ΔPBR in MCF7 cells inducibly expressing GFP-DLC3. E-cadherin-specific immunostainings. Images are maximum intensity projections of several confocal sections. Scale bars: 10 μm. (E and F) Analysis of images from (D). Graph shows the mean fluorescence intensity (MFI ±SEM) of the signal at cell junctions versus the cytoplasmic signal for GFP (E) or E-cadherin (F) (*n* = 3; *N* = 50, 43 cells; t test: *p* = 0.0123 (E), *p* = 0.5194, ns = not significant (F)). (G) Biochemical fractionation of MCF7 cells stably expressing GFP-DLC3 K725E or K725E ΔPBR into soluble supernatant and membrane-containing pellet fractions. Fractions were analyzed by immunoblotting with the indicated antibodies followed by HRP-coupled secondary antibody. (H) Shown is the distribution of GFP signal in the immunoblotted fractions analyzed by Fiji, normalized to GAPDH (supernatant fraction) or transferrin receptor (pellet fraction) (line shows mean of 4 independent experiments; two-way ANOVA with Sidak’s multiple comparison test: *p* = 0.0147).

Binding of cytosolic proteins to phospholipid-rich, negatively charged cellular membranes often involves electrostatic interactions with adjacent clusters of basic amino acids separated by hydrophobic residues.^16^^,^^17^ We therefore bioinformatically screened^18^ the DLC3 sequence for such polybasic domains that could potentially mediate DLC3 membrane binding. The most prominent peak identified by the sequence analysis corresponds to a 23 amino acid stretch within the region between the sterile alpha motif (SAM) and GAP domains (Figure 1A). This region is rich in the basic amino acids arginine, lysine, and histidine and is therefore referred to as polybasic region (PBR) hereafter. Of note, this PBR motif showed a high degree of sequence similarity with orthologous DLC3 sequences from higher mammalian species, hinting at an evolutionary conserved function (Figure S1A). In contrast, screening of the DLC1 and DLC2 sequences for polybasic domains did not show a similar prominent peak in the N-terminal linker region as observed for DLC3. Instead a previously reported PBR,^19^ located more C-terminally, directly adjacent to the GAP domain and conserved in all DLC family members was identified as top hit (Figure S1B). Moreover, sequence alignment revealed that the aminoterminal DLC3 PBR was only partially conserved in DLC1 and DLC2 and harbored several additional basic residues (Figure S1C). These analyses hint at a unique role of the newly identified PBR for DLC3 regulation.

In order to test a potential function of this DLC3 PBR in membrane binding, we first performed lipid overlay experiments using recombinant GST-tagged aminoterminal DLC3 fragments purified from *E. coli* (Figure S1D). Interestingly, whereas the DLC3 fragment including the PBR (aa 2–232) interacted with negatively charged lipids such as phosphoinositides and phosphatidic acid, the fragment lacking the PBR (aa 2–195) failed to do so (Figures 1B and 1C). To study the importance of the PBR in the context of the full-length protein, we generated a PBR deletion mutant (DLC3-ΔPBR) and analyzed its localization by immunofluorescence microscopy in stable MCF7 cells expressing these constructs in a doxycycline-inducible manner. To prevent impairment of epithelial morphology, the GAP-inactive DLC3 K725E point mutant was used for these experiments.^8^ Compared to GFP, the intact GFP-DLC3 protein was enriched at cell-cell contacts marked by E-cadherin (Figures 1D and S1E). By contrast, the DLC3-ΔPBR mutant was significantly more cytosolic and showed no enrichment at E-cadherin-positive regions (Figures 1D and 1E). The ratio of junctional/cytoplasmic E-cadherin signal was similar for cells expressing full-length or DLC3-ΔPBR GFP-DLC3 K725E, indicating that mislocalization of the DLC3-ΔPBR was not due to an impaired integrity of adherence junctions (Figure 1F). Further, assessment of DLC3-ΔPBR mutant localization using alternative markers for cell-cell contacts, including beta-catenin and ZO-1, yielded results comparable to those using E-cadherin staining (Figure S1F). These results were further consistent with biochemical fractionation experiments, which showed that by comparison to the full-length protein, the DLC3-ΔPBR mutant was stronger enriched in the supernatant fraction containing soluble cytosolic proteins (Figures 1G and 1H). These findings suggest that the newly identified PBR is important for the targeting of DLC3 to cellular membranes.

### Phosphorylation of the DLC3 PBR on S208 and S215 regulates membrane binding

**Figure 2:**
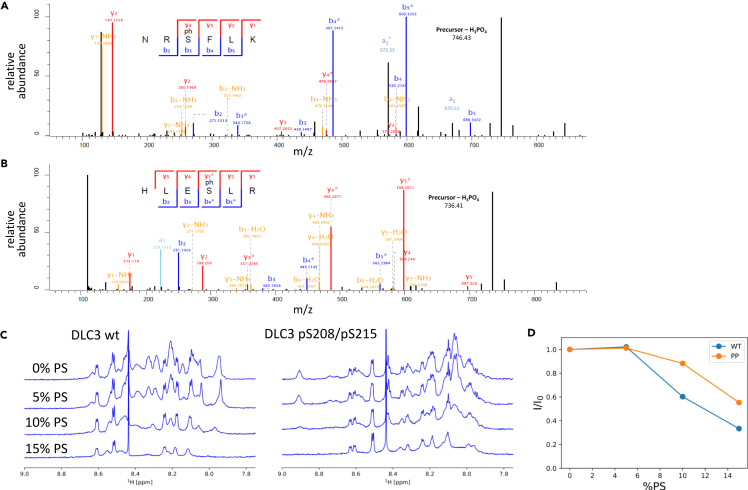
DLC3 PBR phosphorylation impairs membrane interaction *in vitro* (A and B) Fragmentation mass spectra of the phosphopeptides NRpSFLK (A) and HLEpSLR (B) corresponding to amino acids 206–211 and 212–217 in DLC3, respectively, obtained from immunoprecipitated FLAG-tagged DLC3. (C) Superposition of the NMR spectra of 0.1 mM peptides encompassing DLC3 aa 199–221 wt (left) or phosphorylated on serines 208 and 215 (right) measured in the presence of unilamellar vesicles containing variable amounts of the negatively charged POPS. From top to bottom: 100% POPC, 5% POPS/95% POPC, 10% POPS/90% POPC, 15% POPS/85% POPC, total lipid concentration 2 mM. The intensity of the NMR signals progressively decrease due to the increasing interaction of the PBR peptide with the negatively charged vesicles. (D) The dependence of the total integral intensity (I) of the NH signals on the POPS percentage in the vesicles (%PS) presented as a ratio to the integral intensity at 0% POPS (I_0_) that quantifies the signal reduction. The signal intensities of the doubly phosphorylated peptide decrease less than the unmodified peptide, indicating reduced interaction with the membrane upon phosphorylation.

Phosphorylation has been proposed to act as an electrostatic switch to control the interaction between polybasic domain proteins and cellular membranes.^17^ Indeed, *in silico* analysis using Scansite4.0 and NetPhos3.1 predicted with high stringency two phosphorylation sites within the DLC3 PBR, at serines 208 and 215. To confirm these predictions, mass spectrometry analysis was performed on ectopically expressed full-length Flag-DLC3. The phosphorylation at both serine residues was unequivocally identified with this approach (Figures 2A and 2B). To examine the functional consequences of PBR phosphorylation we resorted to biophysical assays using small unilamellar vesicles (SUVs) as membrane mimetics, and modified PBR peptides. NMR binding studies were conducted to analyze the interaction between a peptide corresponding the core PBR, either unphosphorylated (wild-type) or phosphorylated (with incorporated phosphoserines) and SUVs containing varying amounts of negatively charged phosphatidylserine (POPS). Negatively charged vesicles clearly affected the NMR spectra of the DLC3 wild-type peptide, demonstrating protein-lipid interaction (Figure 2C left). Specifically, we observed extensive progressing line broadening for the majority of the peptide signals, with only a small number of signals with reduced intensities detected at 15% of POPS. By contrast, the phosphorylated DLC3 peptides showed markedly smaller changes in the spectra at the same POPS concentration (Figure 2C right). Many of the phosphorylated peptide signals were still present in the spectrum at 15% of POPS, and the overall intensity of the signals in the NH-region of the spectrum was significantly higher for the phosphorylated peptide (Figure 2D). This indicates that the phosphorylation of the PBR reduces the interactions of DLC3 with negatively charged membranes.

**Figure 3:**
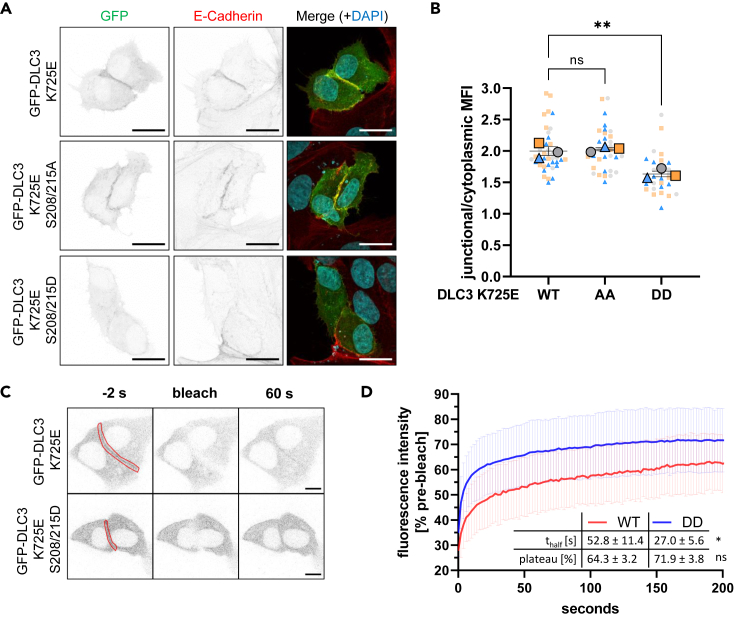
Phospomimetic DLC3 PBR mutants show impaired membrane association *in cellulo* (A) Localization of GFP-DLC3 K725E or phosphodeficient S208/215A or phosphomimetic S208/215D muteins in transiently transfected MCF7 cells. GFP- and E-cadherin-specific immunostainings plus nuclear counterstain (DAPI). Images are maximum intensity projections of several confocal sections. Scale bars: 20 μm. (B) Graph shows the mean fluorescence intensity (MFI ±SEM) of the GFP signal at cell junctions versus the cytoplasmic GFP signal (*n* = 3, *N* = 39, 33, 30); one-way ANOVA with Dunnett’s post-test: WT vs. AA *p* = 0.8765; WT vs. DD *p* = 0.0037). (C and D) Fluorescence recovery [%] after photobleaching cell-cell contact regions of transiently transfected MCF7 cells expressing GFP-DLC3 K725E with wild-type PBR (WT) or phosphomimetic S208/215D mutations (DD). Images show an exemplary site immediately before (−2 s), immediately after (bleach) and 60 s after photobleaching. Red outline indicates photobleached region. Graph shows mean ± SD, *N* = 9, 11 from two independent experiments. Intensity curves were analyzed by one-phase association nonlinear regression to obtain half-time of fluorescence recovery (t_half_) and mobile fraction (plateau) (t test t_half_: *p* = 0.0445; t test plateau: *p* = 0.1485; shown is mean ± SEM).

We next complemented these biophysical findings with fluorescence localization experiments in MCF7 cells overexpressing full-length DLC3 constructs containing PBR mutations. Exchange of serines 208 and 215 to alanine, which cannot be phosphorylated, had no effect on the localization of DLC3, which was still enriched at cell-cell contacts (Figures 3A and 3B). By contrast, mutations to aspartate, mimicking the negative charge introduced by phosphorylation, significantly reduced the proportion of DLC3 localized at cell-cell contacts. These results were confirmed in cells inducibly expressing the constructs, where membrane association of the phosphomimetic mutant was signficantly reduced compared to the phosphodeficient mutant (Figure S2A), while E-cadherin distribution was not altered (Figure S2B). Additionally, similar differences in localization between the mutants were observed when co-staining cells expressing the GFP-DLC3 constructs for beta-catenin and ZO-1 (Figure S2C). Moreover, in FRAP analyses, the phosphomimetic DLC3 mutant exhibited a significantly faster recovery of fluorescence after photobleaching at cell-cell contacts compared to the protein with an unaltered PBR sequence (Figures 3C, 3D, and S2D), hinting at a higher turnover at or facilitated detachment from the membrane. Taken together, these data point toward a phosphoregulatory switch in the DLC3 PBR that regulates its membrane binding capacity.

### A PBR-dependent role for DLC3 in the regulation of cell division

**Figure 4:**
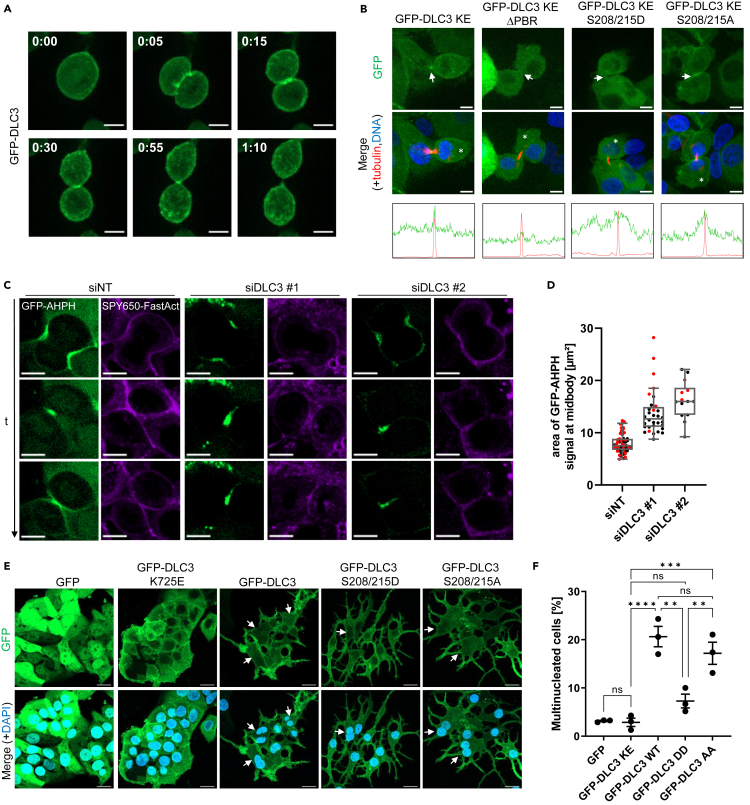
A PBR-dependent role for DLC3 in the regulation of cell division (A) Expression of GFP-DLC3 in stable MCF7 cells was induced for 24 h with doxycycline and cells were analyzed by live-cell imaging. Time stamp: h:mm, scale bars: 10 μm. (B) Expression of indicated GFP-tagged DLC3 constructs (green) in stable MCF7 cells was induced for 24 h with doxycycline and cells were analyzed by live-cell imaging. Midbodies, indicated by arrows, were identified using SPY555-tubulin staining (red). Nuclei were counterstained with SPY650-DNA. Line plots show mean fluorescence signal along the perimeter of cells marked with asterisks. Scale bars: 10 μm. (C) MCF7 cells stably expressing the Rho-GTP biosensor GFP-AHPH (green) were transfected with the indicated siRNAs. After 72 h, cells were stained with SPY650-FastAct (magenta) and analyzed by live-cell imaging. Representative maximum intensity projections of selected time frames from live-cell imaging movies are shown. Scale bars: 10 μm. (D) The area of GFP-AHPH sensor signal in cells from (C) at the midbody area was quantified with Fiji (*n* = 2; *N* = 57, 32, 14) and normalized to control siRNA. Graph shows individual sample points and means in a boxplot with Tukey whiskers. (E) Expression of indicated GFP-tagged DLC3 constructs in stable MCF7 cells was induced for 72 h with doxycycline. Cells were fixed, nuclei were counterstained with DAPI and samples analyzed by fluorescence microscopy. Multinucleated cells are marked with an arrow. Scale bars: 20 μm. (F) The percentage of multinucleated cells was determined manually. Graph shows the means (±SEM) of three independent experiments (*n* = 3; *N* = 318, 324, 189, 173, 183). KE: GAP-inactive K725E mutation, WT: wild-type, DD: phosphomimetic S208/215D mutations, AA: phosphodeficient S208/215A mutations. One-way ANOVA with Tukey’s post-test: GFP vs. KE *p* = 0.99994; KE vs. WT *p* = 0.00009; KE vs. DD *p* = 0.34386; KE vs. AA *p* = 0.00058; WT vs. DD *p* = 0.00100; WT vs. AA *p* = 0.55891; DD vs. AA *p* = 0.00875.

In order to gain deeper insights into the functional role of the PBR in the dynamic regulation of DLC3 localization, we performed long-term live-cell imaging experiments with MCF7 cells expressing the GFP-DLC3 variants of interest in an inducible manner. Remarkably, we observed a distinctive pattern of DLC3 localization in dividing cells, where toward the end of cytokinesis the protein accumulated around a transient structure likely representing the midbody (Figure 4A, Video S1), a structure important for the final separation of daughter cells. Using a live-cell tubulin dye to stain the midbody (Figure S3), we found that this specific localization was not dependent on the GAP activity of DLC3 (Figure 4B, Video S2), while it was absent in the PBR deletion (Figure 4B, Video S3) and phosphomimetic PBR mutants (Figure 4B, Video S4). The phosphodeficient mutant showed no alteration (Figure 4A, Video S5).

Video S1. GFP-DLC3 localizes to the midbody region during cell division, related to Figure 4A Expression of GFP-tagged DLC3 wild-type in stable MCF7 cells was induced for 24 h with doxycycline and cells were analyzed by live-cell imaging. Shown is a representative cell division event. Timestamp: hh:mm, scale bar: 10 μm.

Video S2. GFP-DLC3 K725E localizes to the midbody region during cell division, related to Figure 4B Expression of GFP-tagged DLC3 K725E in stable MCF7 cells was induced for 24 h with doxycycline and cells were stained with SPY555-tubulin and SPY650-DNA and analyzed by live-cell imaging. Shown is a representative cell division event. Left: GFP-DLC3 K725E (green), right: merge with SPY555-tubulin (red) and SPY650-DNA (blue). Timestamp: hh:mm, scale bar: 10 μm.

Video S3. GFP-DLC3 K725E ΔPBR does not localize to the midbody region during cell division, related to Figure 4B Expression of GFP-tagged DLC3 K725E ΔPBR in stable MCF7 cells was induced for 24 h with doxycycline and cells were stained with SPY555-tubulin and SPY650-DNA and analyzed by live-cell imaging. Shown is a representative cell division event. Left: GFP-DLC3 K725E ΔPBR (green), right: merge with SPY555-tubulin (red) and SPY650-DNA (blue). Timestamp: hh:mm, scale bar: 10 μm.

Video S4. The phosphomimetic GFP-DLC3 K725E S208/215D mutant does not localize to the midbody region during cell division, related to Figure 4B Expression of GFP-tagged DLC3 K725E S208/215D in stable MCF7 cells was induced for 24 h with doxycycline and cells were stained with SPY555-tubulin and SPY650-DNA and analyzed by live-cell imaging. Shown is a representative cell division event. Left: GFP-DLC3 K725E S208/215D (green), right: merge with SPY555-tubulin (red) and SPY650-DNA (blue). Timestamp: hh:mm, scale bar: 10 μm.

Video S5. The phosphodeficient GFP-DLC3 K725E S208/215A mutant localizes to the midbody region during cell division, related to Figure 4B Expression of GFP-tagged DLC3 K725E S208/215A in stable MCF7 cells was induced for 24 h with doxycycline and cells were stained with SPY555-tubulin and SPY650-DNA and analyzed by live-cell imaging. Shown is a representative cell division event. Left: GFP-DLC3 K725E S208/215A (green), right: merge with SPY555-tubulin (red) and SPY650-DNA (blue). Timestamp: hh:mm, scale bar: 10 μm.

Rho GTPases play a critical role in the coordination of cytokinesis, by regulating the formation of the contractile actomyosin ring, the ingression of the cleavage furrow, and the formation and resolution of the midbody.^20^ To test whether endogenous DLC3 is involved in the regulation of Rho activity during cytokinesis, we performed live cell imaging experiments with MCF7 cells expressing a fluorescent Rho-GTP biosensor (GFP-AHPH) derived from anillin.^21^ As expected, the biosensor signal was enriched at the cleavage furrow and the midbody (Figure 4C, Video S6). DLC3 depletion using two independent siRNAs, as validated by qPCR (Figure S4A), resulted in a stronger enrichment of the GFP-AHPH signal at F-actin positive structures around the midbody (Figures 4B and 4C, Videos S7 and S8). To obtain a better insight into Rho activity dynamics during the course of cytokinesis, cells were stained with live tubulin and DNA dyes, which allowed for the temporal alignment of dividing cells and specific recognition of the midbody (Figure S4B). In agreement with previous data, DLC3 depletion resulted in a significant expansion of the active Rho biosensor signal specifically at the midbody area, as quantified by the mean area of biosensor signal around the midbody (Figure S4C, Videos S9 and S10). However, the mean fluorescence intensity of the Rho biosensor was not significantly altered (Figure S4D). These findings are indicative of altered spatial Rho signaling dynamics during the abscission of dividing cells upon DLC3 depletion. Dysregulation of Rho GTPase activity during cell division can result in cytokinesis defects.^22^^,^^23^^,^^24^ To explore the potential involvement of DLC3 and its PBR-dependent membrane association in the regulation of cytokinesis fidelity, we quantified the number of multinucleated cells observed at 72 h post-induction of the different DLC3 constructs. While cells expressing GAP-inactive GFP-DLC3 K725E exhibited a phenotype similar to GFP-control cells, the expression of wild-type DLC3 resulted in a significant increase in the percentage of multinucleated cells (Figures 4E and 4F). Notably, the GAP-competent phosphodeficient PBR mutant, like the wild-type protein, induced multinucleation, whereas the phosphomimetic PBR mutant did not exhibit this effect. Importantly, western blot analysis confirmed comparable expression levels of the phospho-mutant proteins (Figures S4E and S4F), ruling out differential expression as a cause for the observed differences in the multinucleation phenotype. This finding suggests that abundant localization of DLC3 at cytokinetic structures, depending on PBR phosphorylation, can impair accurate cell division in a GAP-dependent manner. Taken together, these results point toward a novel role of DLC3 in the control of local Rho activity during cytokinesis that relies on the dynamic interaction of DLC3 with membranes via its PBR.

Video S6. GFP-AHPH Rho biosensor localization during cell division, related to Figure 4C MCF7 cells stably expressing the active Rho biosensor GFP-AHPH were transfected with control siRNA. After 72 h, cells were stained with SPY650-FastAct and analyzed by live-cell imaging. Shown is a representative cell division event. Left: GFP-AHPH, right: SPY650-FastAct. Timestamp: hh:mm, scale bar: 10 μm.

Video S7. DLC3 depletion leads to increased Rho activity at the midbody, related to Figure 4C MCF7 cells stably expressing the active Rho biosensor GFP-AHPH were transfected with siRNA DLC3 #1. After 72 h, cells were stained with SPY650-FastAct and analyzed by live-cell imaging. Shown is a representative cell division event. Left: GFP-AHPH, right: SPY650-FastAct. Timestamp: hh:mm, scale bar: 10 μm.

Video S8. DLC3 depletion leads to increased Rho activity at the midbody, related to Figure 4C MCF7 cells stably expressing the active Rho biosensor GFP-AHPH were transfected with siRNA DLC3 #2. After 72 h, cells were stained with SPY650-FastAct and analyzed by live-cell imaging. Shown is a representative cell division event. Left: GFP-AHPH, right: SPY650-FastAct. Timestamp: hh:mm, scale bar: 10 μm.

Video S9. DLC3 depletion leads to increased Rho activity at the midbody, related to Figure S3B MCF7 cells stably expressing the active Rho biosensor GFP-AHPH were transfected with control siRNA. After 72 h, cells were stained with SPY555-tubulin and SPY650-DNA and analyzed by live-cell imaging. Shown is a representative cell division event. Left: GFP-AHPH (green), right: merge with SPY555-tubulin (red) and SPY650-DNA (blue). Timestamp: hh:mm, scale bar: 10 μm.

Video S10. DLC3 depletion leads to increased Rho activity at the midbody, related to Figure S3B MCF7 cells stably expressing the active Rho biosensor GFP-AHPH were transfected with siRNA DLC3 #2. After 72 h, cells were stained with SPY555-tubulin and SPY650-DNA and analyzed by live-cell imaging. Shown is a representative cell division event. Left: GFP-AHPH (green), right: merge with SPY555-tubulin (red) and SPY650-DNA (blue). Timestamp: hh:mm, scale bar: 10 μm.

## Discussion

Given the important role of DLC3 in the maintenance of apical-basal cell polarity and endosomal trafficking, a tight regulation of its membrane association and proximity to the target Rho GTPase is necessary. By analyzing the DLC3 protein sequence, we here identified a novel PBR within its amino terminal region that facilitates the binding of DLC3 to lipids and negatively charged SUVs *in vitro* and to cell-cell contacts in epithelial cells. Within the PBR, we furthermore identified two phosphorylation sites that regulate the association of DLC3 with membranes. Protein phosphorylation is a fast and reversible post-translational modification ideally suited to regulate membrane binding dynamics. The addition of a negatively charged phosphate group changes the local electrostatic environment and can further induce a more stable, ordered conformation, both of which may result in impaired membrane binding.^25^ Indeed, phosphorylation of DLC3 PBR peptides decreased their interaction with negatively charged membrane model systems compared to their unphosphorylated form, which goes in line with a phosphomimetic DLC3 mutant showing impaired association to cell-cell junctions in intact cells. This regulatory mechanism could play a role in dynamic processes such as the maintenance of Rho and Rac gradients during cell polarity and RhoB regulation during endocytic recycling, but also on a larger scale in the rapid repolarization of cells after cytokinesis or during cell migration. These results point toward a phosphoregulatory mechanism controlling the timing of local DLC3 membrane association and Rho signaling. Given the partial protonation of histidine at physiological pH,^26^ DLC3 harbors more than three additional net positive charges in its aminoterminal PBR over DLC1 and DLC2. This disparity in charge distribution suggests that the PBR may not function similarly well in binding to negatively charged lipids for DLC1 and DLC2, thus representing another unique molecular feature of DLC3 alongside the previously identified PDZ ligand motif.^8^

In this study, we further uncovered a distinct localization pattern of DLC3 during cytokinesis and a hitherto unknown putative involvement in the regulation of Rho activity during this cellular process. The mechanism by which DLC3 localizes to specific structures during cytokinesis remains to be explored, as neither Scribble nor SNX27, previously associated with the recruitment of DLC3 to other localizations,^8^^,^^10^ have been linked to cytokinesis. Patterned zones of RhoA activation at sites of furrow formation are required for the assembly of the contractile actomyosin ring that constricts and leads to cleavage furrow formation.^27^ In this context, regulation of RhoA activity was reported to be mediated by the GEF Ect2 and the GAP p190GAP, respectively.^28^^,^^29^ In addition, a circuit involving Rho, Ect2 and the GAP RGA-3/4 associated with the cleavage furrow during cytokinesis in frog and starfish was recently described.^30^ As furrow formation and ingression appeared to proceed normally in DLC3 depleted cells, an involvement in Rho regulation at this stage seems unlikely. In the late stages of cleavage furrow ingression, the spindle microtubules are bundled into the midbody, a transient bridge connecting the two daughter cells. Components of the contractile ring including RhoA are retained at this structure to execute the final phase of cell abscission.^31^^,^^32^ However, previous experiments using a constitutively active mutant suggest that RhoA must first undergo inactivation to accumulate at the midbody.^32^ We observed enrichment of GFP-tagged DLC3 at distinct structures in the midbody region in a PBR-dependent manner. This localization appears to be subject to the same phosphoregulatory mechanism as a phosphomimetic mutant failed to show such localization patterns. Consequently, the inducible expression of exogenous DLC3 variants resulted in an increase in multinucleated cells, depending on the GAP activity and mutations of the PBR phosphorylation sites. This phenotype is directly linked to aberrant abscission^33^ and mimics the effects reported upon depletion of the Rho activator and known DLC3 antagonist GEF-H1.^23^^,^^34^ Our observations suggest that the prolonged association of overexpressed wild-type DLC3 with the midbody region contributes to cytokinesis defects and multinucleation, whereas the more dynamic membrane interaction of the phosphomimetic mutant does not result in multinucleation. This underscores the critical role of timing DLC3’s function during this essential cellular process. In addition, increased recruitment of the GFP-AHPH Rho activity biosensor around the midbody was observed after DLC3 depletion, further corroborating a role for DLC3 in local Rho regulation at this stage. Of note, the biosensor also integrates activities of the closely related RhoB and RhoC, which were shown to partially compensate for RhoA during cytokinesis.^35^ Recently, it was reported that timely activation of the RhoA homolog in yeast is dependent on Cdc42,^36^ toward which DLC3 also shows some GAP activity.^12^^,^^37^ In addition, previous studies have described the binding of specific lipid species to DLC1 through the PBR directly adjacent to the GAP domain and the carboxyterminal StAR (steroidogenic acute regulatory)-related lipid transfer (START) domain, regulating its GAP activity.^19^^,^^38^ While *in vitro* lipid binding of these conserved regions has also been demonstrated for DLC3, their specific impact on the activity of the protein remains unknown. Thus, further research is necessary to elucidate the mechanisms by which DLC3 influences Rho signaling during cytokinesis. Interestingly, atypical protein kinase C (aPKC) family kinases, recognized as master regulators of cell polarity,^39^ were reported to exhibit high activity toward PBR-containing substrates,^40^ and have been implicated in cytokinesis regulation.^41^ Our preliminary results show that aPKC is able to phosphorylate DLC3 on serines 208 and 215 *in vitro* (Figure S5). Whether aPKC or other mitotic kinases, such as Plk1 or Aurora kinases, are involved in DLC3 phosphorylation during cytokinesis should be addressed in future studies. As the activity of many kinases is highly dysregulated during carcinogenesis,^42^ the discovery of the membrane-binding region and phosphorylation-dependent localization switch in this study opens new perspectives on the potential functional inactivation of DLC3 and how it may contribute to cytokinesis defects in cancer cells.

### Limitations of the study

Our study presents important insights into the regulation of DLC3’s membrane association and its potential role in cytokinesis. However, our findings, primarily based on *in vitro* assays and cell culture experiments, need validation in more complex physiological settings. The identity of the upstream signaling pathways and specific kinases responsible for phosphorylating DLC3 remain to be elucidated. Additionally, while we observe DLC3 localization around the midbody, where it regulates Rho GTPase turnover, DLC3 is not exclusively enriched at the midbody during cytokinesis. Live cell imaging experiments have revealed that GFP-DLC3 accumulates at the ingressing cleavage furrow and persists there until abscission. Future research should explore the kinetics of DLC3 membrane association during cytokinesis and how this localization correlates with GAP activity and Rho GTPase turnover.

## STAR★Methods

### Key resources table

**Table:** 

REAGENT or RESOURCE	SOURCE	IDENTIFIER
**Antibodies**		

mouse anti-α-tubulin mAb	Sigma-Aldrich	Cat# 05–829; RRID: AB_310035
mouse anti-FLAG M2 mAb	Sigma-Aldrich	Cat# F1804; RRID: AB_262044
rabbit anti-GAPDH pAb	Sigma-Aldrich	Cat# G9545; RRID: AB_796208
mouse anti-transferrin receptor mAb	Invitrogen	Cat# 13-6800; RRID: AB_2533029
goat anti-GST pAb	GE Healthcare	Cat# GE27-4577-01; RRID: AB_771432
mouse anti-GFP mAb	Roche Biosciences	Cat# 11814460001; RRID: AB_390913
mouse anti-β-catenin mAb	BD Biosciences	Cat# 610154; RRID: AB_397555
rabbit anti-E-cadherin mAb	Cell Signaling Technologies	Cat# 3195; RRID: AB_2291471
rabbit anti-ZO-1 mAb	Cell Signaling Technologies	Cat# 13663; RRID: AB_2798287
rabbit anti-GFP mAb	Cell Signaling Technologies	Cat# 2956; RRID: AB_1196615

**Bacterial and virus strains**		

*Escherichia coli* BL21 (DE3)	Thermo Fisher	Cat# EC0114
*Escherichia coli* DH5α	Thermo Fisher	Cat# 18265017
*Escherichia coli* Stbl3	Thermo Fisher	Cat# C737303

**Chemicals, peptides, and recombinant proteins**		

DLC3 PBR peptides	ChinaPeptides Co., Ltd.	N/A
SPY650-FastAct	Spirochrome	Cat# SC505
SPY555-tubulin	Spirochrome	Cat# SC203
SPY650-DNA	Spirochrome	Cat# SC501
Recombinant GST-PKCζ	SignalChem Biotech	Cat# P75-10G

**Critical commercial assays**		

Membrane lipid strips	Echelon Biosciences	Cat# P-6002

**Deposited data**		

Mass spectrometry proteomics data	ProteomeXchange Consortium	PXD045808

**Experimental models: Cell lines**		

HEK293T	ATCC	Cat# CRL-3216
Lenti-X 293T	Philipp Rathert, Institute of Biochemistry and Technical Biochemistry, University of Stuttgart, Germany	N/A
MCF7	Cornelius Knabbe, Institute of Clinical Pharmacology, Stuttgart, Germany	N/A
MCF7 stably expressing GFP-DLC3 K725E	This paper	N/A
MCF7 stably expressing GFP-DLC3 K725E ΔPBR	This paper	N/A
MCF7 stably expressing GFP-AHPH	This paper	N/A
MCF7 inducibly expressing GFP-DLC3 wt	This paper	N/A
MCF7 inducibly expressing GFP-DLC3 S208/215A	This paper	N/A
MCF7 inducibly expressing GFP-DLC3 S208/215D	This paper	N/A
MCF7 inducibly expressing GFP-DLC3 K725E	This paper	N/A
MCF7 inducibly expressing GFP-DLC3 K725E ΔPBR	This paper	N/A
MCF7 inducibly expressing GFP-DLC3 K725E S208/215A	This paper	N/A
MCF7 inducibly expressing GFP-DLC3 K725E S208/215D	This paper	N/A

**Oligonucleotides**		

Primers for cloning and site-directed mutagenesis	This paper (see Table S1)	N/A
Primers for quantitative PCR	This paper (see Table S1)	N/A
ON-TARGETplus Non-targeting Pool siRNA	Dharmacon	Cat# D-001810–10
Silencer Select DLC3 siRNA	ambion life technologies	Cat# s18826
siGENOME SMARTpool human DLC3	Dharmacon	Cat# M-010254-00-0010

**Recombinant DNA**		

pEGFP-DLC3	Holeiter et al.^13^	N/A
pEGFP-DLC3 K725E	Holeiter et al.^13^	N/A
pGEX-6P3-GST-DLC3-(2-195)	This paper	N/A
pGEX-6P3-GST-DLC3-(2-232)	This paper	N/A
pGEX-6P3-GST-DLC3-(2-232) S208A	This paper	N/A
pGEX-6P3-GST-DLC3-(2-232) S215A	This paper	N/A
pGEX-6P3-GST-DLC3-(2-232) S208/S215A	This paper	N/A
pEGFP-DLC3 ΔPBR	This paper	N/A
pEGFP-DLC3 K725E ΔPBR	This paper	N/A
pEGFP-DLC3 K725E S208/215A	This paper	N/A
pEGFP-DLC3 K725E S208/215D	This paper	N/A
pCW57-EGFP-DLC3	This paper	N/A
pCW57-EGFP-DLC3 S208/215A	This paper	N/A
pCW57-EGFP-DLC3 S208/215D	This paper	N/A
pCW57-EGFP-DLC3 K725E	This paper	N/A
pCW57-EGFP-DLC3 K725E ΔPBR	This paper	N/A
pCW57-EGFP-DLC3 K725E S208/215A	This paper	N/A
pCW57-EGFP-DLC3 K725E S208/215D	This paper	N/A
pEGFPC1-AHPH	Priya et al.^21^	N/A
pLV-EGFP-AHPH	This paper	N/A

**Software and algorithms**		

BH search	Brzeska et al.^18^	https://helixweb.nih.gov/bhsearch/
Scansite 4.0	Obenauer et al.^43^	https://scansite4.mit.edu
NetPhos 3.1	Blom et al.^44^	https://services.healthtech.dtu.dk/services/NetPhos-3.1/
Fiji	Schindelin et al.^45^	https://fiji.sc/
Zen 3.6 blue edition	Zeiss	N/A

### Resource availability

#### Lead contact

All requests for further information and resources/reagents should be directed to the lead contact, Monilola Olayioye (monilola.olayioye@izi.uni-stuttgart.de).

#### Materials availability

All unique reagents generated in this study will be made available from the lead contact (M.A.O.) and may require a completed materials transfer agreement.

#### Data and code availability

• The mass spectrometry proteomics data have been deposited to the ProteomeXchange Consortium via the PRIDE partner repository with the dataset identifier PXD045808.
• This study did not generate new unique code.
• Any additional information required to reanalyze the data reported in this paper is available from the lead contact upon request.

### Experimental model and study participant details

#### Cell lines

Cultured cell lines used in this study were grown in RPMI 1640 (ThermoFisher) supplemented with 10% FCS without antibiotics under sterile conditions in a humidified atmosphere of 5% CO_2_ at 37°C. HEK293T cells were obtained from ATCC (Manassas, USA), MCF7 cells were kindly provided by Cornelius Knabbe (Institute of Clinical Pharmacology, Stuttgart, Germany). All cell lines were authenticated by STR analysis and regularly tested for mycoplasma contamination. MCF7 cells were transiently transfected using Lipofectamine LTX with Plus Reagent (Invitrogen) according to the manufacturer’s instructions. Lenti-X 293T cells were kindly provided by Philipp Rathert (Institute of Biochemistry and Technical Biochemistry, University of Stuttgart, Germany). Plasmid transfection of HEK293T or Lenti-X cells was performed using a 1:3 (w/w) mixture of DNA to polyethylenimine (Sigma Aldrich). For production of lentivirus, Lenti-X cells were transfected with the lentiviral expression constructs and the packaging plasmid psPAX2 and the envelope plasmid pCMV-VSV-G. Stable expression cell lines were generated by selection with 1 mg/ml G418 (Carl Roth, Karlsruhe, Germany) after transient transfection or lentiviral transduction. For RNAi, cells were transfected with siRNA for 72 h using Lipofectamine RNAiMAX (Invitrogen) according to manufacturer’s instructions.

### Method details

#### Antibodies and reagents

The following antibodies were used in this study: mouse anti-α-tubulin mAb (used 1:10000 in WB, 05–829), mouse anti-FLAG M2 (1:1000 in WB, F1804) and rabbit anti-GAPDH pAb (1:15000 in WB, G9545) from Sigma-Aldrich (St.Louis, USA); mouse anti-transferrin receptor mAb (used 1:1000 in WB, 13-6800) from Invitrogen (Karlsruhe, Germany); goat anti-GST pAb (1:5000 in WB, GE27-4577-01) from GE Healthcare (Piscataway, USA); mouse anti-GFP mAb (1:250 in IF, 1:1000 in WB, 11814460001) from Roche Biosciences (Basel, Switzerland); mouse anti β-catenin mAb (1:500 in IF, 610154) from BD Biosciences (Franklin Lakes, USA); rabbit anti-E-cadherin mAb (1:200 in IF, 3195), rabbit anti-ZO-1 mAb (1:200 in IF, 13663) and rabbit anti-GFP mAb (1:1000 in WB, 2956) from Cell Signaling Technologies (Danvers, USA). HRP-labeled secondary goat anti-mouse and anti-rabbit IgG antibodies were purchased from Dianova (Hamburg, Germany), Alexa-Fluor-labeled secondary IgG antibodies were from Invitrogen. DAPI was from Sigma-Aldrich (1:5000 in IF). SPY650-FastAct, SPY555-tubulin and SPY650-DNA were purchased from Spirochrome (Stein am Rhein, Switzerland) and used according to the manufacturer’s instructions. The following siRNAs were used: non-targeting control siRNA (siNT, ON-TARGETplus Non-targeting Pool, D-001810–10; Dharmacon, Lafayette, CO), siDLC3 #1 (Silencer Select DLC3 s18826; ambion life technologies), siDLC3 #2 (siGENOME SMARTpool human DLC3 M-010254-00-0010; Dharmacon). These siRNAs have been extensively characterized in previous studies.^8^^,^^10^^,^^11^

#### DNA constructs and cloning

The expression constructs pEGFP-DLC3 and pEGFP-DLC3 K725E have been described previously.^7^ All oligonucleotides were purchased from Eurofins (Ebersberg, Germany), with sequences listed in Table S1. pEGFP-DLC3-(2-232) was generated by PCR amplification using pEGFP-DLC3 as a template and DLC3 aa2-232 forward and reverse primers. pEGFP-DLC3-(2-195) was generated by PCR amplification using pEGFP-DLC3 as a template and DLC3 aa2-195 forward and reverse primers. The PCR products were cloned into the pEGFP-C1 vector by EcoRI restriction, respectively. pGEX-6P3-GST-DLC3-(2-195) and -(2-232), were subcloned from the corresponding pEGFP-DLC3 constructs by EcoRI restriction into the pGEX-6P3 vector. To generate the pEGFP-DLC3 ΔPBR and pEGFP-DLC3 K725E ΔPBR constructs, the amino acid stretch 196 – 232 was excised by site-directed PCR mutagenesis using the full-length pEGFP-DLC3 construct as a template and DLC3 ΔPBR forward and reverse primers. Phosphodeficient S208A and S215A mutations or phosphomimetic S208D and S215D mutations were generated by site-directed PCR mutagenesis using the S208A, S215A, S208D or S215D forward and reverse primers with the corresponding DLC3 expression constructs as templates, respectively. Doxycyclin-inducible lentiviral GFP-DLC3 expression constructs were generated by digestion of the respective pEGFP-DLC3 constructs with NsiI, NheI, Cfr9I, and subsequent ligation of the GFP-DLC3 fragment in pCW57-MCS1-P2A-MCS2 (Neo) (a gift from Adam Karpf; Addgene plasmid #89180) digested with NheI, BshTI. The pEGFPC1-AHPH plasmid encoding the Rho-GTP biosensor was a kind gift from Alpha Yap (University of Queensland, Australia).^21^ To generate a lentiviral Rho-GTP biosensor expression construct, the pLV-EF1a-IRES-Blast (a gift from Tobias Meyer; Addgene plasmid #85133^46^) vector was digested with EcoRI and BamHI, the insert was amplified by PCR using the EGFP-AHPH forward and reverse primers, and fragments were assembled using NEBuilder HiFi DNA Assembly (NEB). psPAX2 was a gift from Didier Trono (Addgene plasmid #12260), pCMV-VSV-G was a gift from Bob Weinberg (Addgene plasmid #8454^47^). The default *E. coli* strain used for plasmid propagation was DH5α, for lentiviral constructs Stbl3 were used. All constructs were verified by Sanger sequencing (Microsynth Seqlab, Göttingen, Germany).

#### Production of recombinant GST-DLC3 fusion proteins

*Escherichia coli* BL21 (DE3) were transformed with the respective pGEX expression vectors. Expression was induced with 0.5 mM IPTG for 4 h at 37°C. Bacteria were harvested and resuspended in PBS containing Complete protease inhibitor (Roche Diagnostics). After sonification, Triton X-100 was added to a final concentration of 1% (v/v) and lysates were incubated on ice for 15 min. GST-fusion proteins were purified from clarified lysate with glutathione sepharose 4B (GE Healthcare) and beads were washed three times with PBS containing Complete protease inhibitor. For elution of GST-fusion proteins, beads were incubated with elution buffer (50 mM Tris, 10 mM reduced gluthatione in ddH2O, pH 8.0).

#### Protein-lipid overlay assay

According to the manufacturer's instructions, membrane lipid strips (P-6002, Echelon Biosciences, Salt Lake City, USA) were blocked for 1 h in 0.1% (v/v) Tween-20 in PBS containing 3% (w/v) fatty acid-free BSA. Equal volumes of eluted recombinant GST-DLC3 fusion proteins (100 μl) were added to 5 ml of blocking buffer and incubated on the membranes at room temperature for 1.5 h. Following washing with 0.1% (v/v) Tween-20 in PBS, membranes were incubated with anti-GST antibody and subsequently HRP-coupled secondary antibody. Following further washing steps, bound GST-tagged protein was visualized with an enhanced chemiluminescence detection system.

#### Immunofluorescence microscopy and image analysis

Cells grown on glass coverslips coated with 10 μg/ml collagen R (Serva, Heidelberg, Germany) were fixed with 4% PFA in PBS for 15 min at RT. After washing with PBS the samples were incubated in 150 mM glycine in PBS for 15 min and then permeabilized with 0.2% Triton-X-100 in PBS for 5 min and blocked with 5% goat serum in PBS containing 0.1% Tween-20 for 30 min. Samples were incubated with specific primary antibodies diluted in blocking buffer for 2 h at RT, followed by incubation with AlexaFluor® (488, 546) labeled secondary antibodies and DAPI in blocking buffer for 1 h at RT. Coverslips were mounted in Fluoromount-G® (SouthernBiotech; Birmingham, USA) and analyzed at RT on a LSM710 confocal laser scanning microscope or a LSM980 Airyscan 2 (Carl Zeiss, Oberkochen, Germany) equipped with a Plan-Apochromat 63x/1.40 DIC (Carl Zeiss) oil immersion objective using 405-, 488-, 561-nm laser excitation. For each set of replicates, images were acquired with the same laser and confocal settings. Maximum intensity projections, linear adjustments of brightness and contrast, and analysis of mean fluorescence intensity (MFI) of junctional and cytoplasmic regions of interest (ROI) was performed with the ZEN software (Zeiss).

#### Live cell microscopy and image analysis

Cells were seeded onto collagen-coated 35 mm high glass bottom μ-Dishes (ibidi, Gräfelfing, Germany) and analyzed on an AxioObserver microscope (Carl Zeiss) equipped with a CSU-X1 spinning disk module, a Photometrix Evolve 512 EMCCD camera at 37°C and 5% CO_2_. For staining of actin, fresh medium supplemented with SPY650-FastAct (Spirochrome, Stein am Rhein, Switzerland) was added 1 h before imaging started. Cells were imaged using a Plan-Apochromat 63x/1.40 DIC (Carl Zeiss) oil immersion objective using 488- and 638-nm laser excitation. Quantification of the GFP-AHPH signal at the midbody was performed manually with Fiji. For co-staining of tubulin and DNA, fresh media supplemented with SPY555-tubulin and SPY650-DNA (Spirochrome) was added 1 h before live cell imaging. Cells were imaged with a Plan-Apochromat 40x/1.40 DIC (Carl Zeiss) oil immersion objective using 488-, 553- and 638-nm laser excitation. In Fiji, for line plot analysis of fluorescence signals, cell perimeters were traced with the freehand line tool with a width of 3 pixels and intensities measured with the plot profile function. To quantify the GFP-AHPH signal at the midbody for a given timepoint, the midbody ROI was defined by enlarging the tubulin mask by 1 μm. Here, the processed GFP-AHPH signal was thresholded and the resulting mask used to measure the area and MFI in the original image. For FRAP experiments, a UGA-42-Firefly point scanning device with a 473 nm laser (Rapp Optoelectronics, Wedel, Germany) was used. After the acquisition of multiple pre-bleach images, ROIs of GFP-DLC3 signal at cell-cell contacts were bleached separately. Fluorescence recovery was recorded for at least 300 s post-bleach in 2 s intervals and mean fluorescence intensities in the ROI measured using the ZEN software (Zeiss). Individual fluorescence recovery curves were analyzed by one-phase association nonlinear regression with GraphPad Prism.

#### Cell lysis, cellular fractionation and immunoblotting

Separation of the cytoplasmic fraction (=supernatant) and the detergent-soluble fraction (=pellet) was modified from a previously described protocol.^8^ To this end, 5 x 10^6^ MCF7 cells were seeded in 10 cm dishes and treated with doxycycline 24 h later. The cells were lysed 24 h after induction of the various GFP-DLC3 constructs. To separate the cytoplasmic from the detergent-soluble fraction, the cells were resuspended in 750 μL detergent-free fractionation buffer [50 mM Hepes (pH 7.4), 100 mM NaCl, 5 mM MgCl_2_, 5 mM EDTA, 1 mM DTT, 0.5 mM PMSF, 1 mM Na_3_VO_4_, 10 mM sodium fluoride, 20 mM β-glycerophosphate, Complete protease inhibitor cocktail without EDTA (Roche)]. After 10 min incubation on ice, the samples were subjected to four freeze/thaw cycles in liquid nitrogen, clarified by centrifugation for 20 min at 16,000 *g*, 4°C and the supernatant was collected. The pellet was washed once with fractionation buffer and then solubilized in 750 μL modified RIPA buffer [50mM Hepes (pH 7.4), 150 mM NaCl, 5 mM MgCl_2_, 5 mM EDTA, 1 mM DTT, 1% Triton-X-100, 0.5% sodium deoxycholate, 0.5 mM PMSF, 1 mM Na_3_VO_4_, 10 mM sodium fluoride, 20 mM β-glycerophosphate, Complete protease inhibitor cocktail without EDTA (Roche)] by sonication with an EpiShear Probe Sonicator (Active Motif). Each sample was sonicated with 2 pulses of 20 s at an amplitude of 40%, a pause of 30 s was used after each pulse. Subsequently, the samples were incubated at 4°C for 20 min while rolling end over end, followed by centrifugation for 10 min at 16,000 *g* and 4°C. The supernatant was collected as the membrane fraction (=pellet). Whole cell lysates were prepared by lysing the cells in the modified RIPA buffer, sonication and clearing as described above for the pellet fraction. Equal volumes of each fraction were analyzed by immunoblotting. GFP-DLC3 distribution in the fractions was determined after normalizing the GFP signal to the soluble protein GAPDH or the membrane protein transferrin receptor for the cytoplasmic and pellet fractions, respectively. Proteins were separated by SDS-PAGE and transferred to nitrocellulose membranes (iBlot Gel Transfer Stacks; Invitrogen). Blots were blocked with 0.5% blocking reagent (Roche) in PBS containing 0.05% Tween-20 and incubated with specific primary antibodies, followed by HRP-labeled secondary antibodies for detection with ECL substrates (ThermoFisher).

#### NanoLC-MS/MS analysis and MS data processing

HEK293T cells expressing FLAG-DLC3 were lysed in 1% TEB buffer (RIPA buffer without sodium deoxycholate and SDS). Flag-tagged proteins were immunoprecipitated from cell lysates with Flag M2 agarose (Sigma Aldrich). Beads were resuspended in denaturation buffer (6 M urea, 2 M thiourea, 10 mM Tris buffer, pH 8.0), and proteins were digested on beads with LysC or trypsin as described previously.^48^ Acidified peptides were purified via PHOENIX Peptide Cleanup Kit (PreOmics) according to user manual.

Phosphopeptide enrichment was done using TiO_2_ beads (Sachtopore NP 5 μm, 300 A, SNX 030S005, Huntsmen Corporation). Beads were resuspended in DHB solution (80% ACN, 1% TFA, 3% 2,5-dihydroxybenzoic acid (DHB)) and incubated for 20 min. After its removal 90% of purified peptides dissolved in 80% acetonitrile (ACN)/6% *trifluoroacetic acid* (TFA) were added to TiO_2_ beads (beads to protein ratio, 1:2) and incubated for 10 min. Bound phosphopeptides were washed first with 30% ACN/1% TFA, followed by 80% ACN/1% TFA. Elution from the beads was performed with 5% NH_4_OH in 60% ACN in a first step, and with 80% ACN/1% formic acid (FA) in a second round. Acidified eluates were pooled, and after evaporation of organic solvent they were further purified by C18 StageTips.^49^ Peptides were subjected to two consecutive rounds of enrichment.

Peptides and enriched phosphopeptides were analysed on an EasyLC coupled to a QExactive HF mass spectrometer (both Thermo Fisher Scientific) as described elsewhere^50^ with slight modifications: peptide mixtures were separated using a 57 minute segmented gradient of 10-33-50-90% of HPLC solvent B (80% ACN in 0.1% FA) in HPLC solvent A (0.1% FA) at a flow rate of 200 nl/min. Precursor ions were acquired in the mass range from m/z 200 to 1650 in the Orbitrap mass analyzer at a resolution of 60,000 (fill time 25 ms, AGC target 3x10^6^). In each scan cycle, the seven most intense precursor ions were sequentially fragmented using higher energy collisional dissociation (HCD) fragmentation. In all measurements, sequenced precursor masses were excluded from further selection for 30 s. MS/MS scans were acquired with a resolution of 60,000 (fill time 220 ms, AGC target 10^5^).

MS spectra were processed with MaxQuant software package version 1.5.2.8^51^ with integrated Andromeda search engine.^52^ Database search was performed against a *Homo sapiens* database obtained from Uniprot, containing 96,817 protein entries, the sequence of FLAG-DLC3 and 284 commonly observed contaminants. Endoprotease LysC and trypsin, respectively were defined as proteases with a maximum of three missed cleavages. The minimum peptide length was set to five. Oxidation of methionine, phosphorylation on serine, threonine and tyrosine, methylation on lysine and arginine, and N-terminal acetylation were specified as variable modifications, whereas carbamidomethylation of cysteine was set as fixed modification. Initial maximum allowed mass tolerance was set to 4.5 parts per million (ppm) for precursor ions and 20 ppm for fragment ions. Peptide, protein and modification site identifications were reported at a false discovery rate (FDR) of 0.01, estimated by the target-decoy approach.^53^

#### Preparation of small unilamellar vesicles

Lipid solutions of POPC (PC) and POPS (PS) in chloroform were mixed in the required proportions (0, 5, 10 and 15% PS) and evaporated under a nitrogen stream. The remaining film was dried under vacuum for 2 h and resuspended in in 10 mM MES buffer, pH 6.1. The resulting solution was sonicated on ice for 7 min in 5/25 s on/off cycles to promote formation of SUVs. After spinning down at 21,000 g for 5 min the supernatant containing the SUVs was transferred to a fresh snap-cap micro centrifuge tube and used immediately for the NMR experiments.

#### NMR spectroscopy

Short peptides of the DLC3 PBR (aa 197-223) were ordered from ChinaPeptides Co., Ltd. (Shanghai, China). For the interaction with lipids, 1D spectra for the wild-type and S208/215 phosphorylated peptides at the concentration of 0.1 mM were measured in the presence of 2 mM SUVs with a different POPS/POPC ratio in 10 mM MES buffer, pH 6.1, 5% D_2_O. DSS was used as an external reference standard. NMR spectra were acquired at 15°C on a Bruker Avance III 800 MHz spectrometer equipped with a 5 mm TCI CryoProbe. The relaxation delay for 1D experiments was 1 s. Spectra were processed and analyzed using TopSpin software (version 4.9, Bruker). Peptides were added from 10 mM stock solutions prepared by weight and validated by integration of the distinct ^δ^CH_3_ signals of the three leucine residues.

#### *In silico* sequence analysis

To predict putative membrane binding region in DLC proteins a modified hydrophobicity scale implemented in the BH search program was used.^18^ Alignment of DLC protein sequences and DLC3 orthologue sequences from different species was performed using BLAST (https://blast.ncbi.nlm.nih.gov/Blast.cgi). Candidate phosphorylation sites were predicted using Scansite 4.0 and NetPhos 3.1.^43^^,^^44^

#### *In vitro* kinase assay

Equal amounts of the purified GST-DLC3 proteins were mixed with Kinase Assay Buffer I (SignalChem Biotech Inc.) containing 2 μCi [γ-^32^P]-ATP (Hartmann Analytic, Braunschweig, Germany) and incubated for 15 min at 37°C in the presence of recombinant PKCζ (SignalChem Biotech Inc., P75-10G). Samples were then resolved by SDS-PAGE, transferred to PVDF membranes and the ionizing radiation was recorded on a PhosphoImager (Molecular Dynamics), followed by immunoblotting of the membrane.

#### Quantitative real-time PCR

RNA was isolated from cells using the NucleoSpin RNA kit (Macherey–Nagel) according to manufacturer’s instructions. 100 ng RNA were used for real-time PCR, using the Power SYBR® Green RNA-to-CT 1-Step kit (Thermo Fisher) with the following primers: DLC3-F: CTGGACCAAGTAGGCATCTTCC, DLC3-R: CTCTTCCATGTAGAGGCTCAGG, GAPDH-F: CCCCTTCATTGACCTCAACTA, GAPDH-R: CGCTCCTGGAAGATGGTGAT. Analysis was performed using the CFX96 Touch Real-Time PCR Detection System (Bio-RAD). To analyze the fold change gene expression, the double delta Ct analysis was used (fold change = 2(-ΔΔCt)). GAPDH served as control gene.

### Quantification and statistical analysis

Data are presented as the mean ± SEM or mean ± SD as indicated in the respective figure legend. Where appropriate, data are presented as superplot.^54^ ‘N’ refers to the total number of sample points and ‘n’ to the number of independent experiments. Data were analyzed using GraphPad Prism 9 with the statistical tests and the resulting p-values detailed in the respective figure legends. A p-value below 0.05 was considered statistically significant, statistical significance is indicated as follows: not significant (ns) for p > 0.05, ∗ for p < 0.05, ∗∗ for p < 0.01, ∗∗∗ for p < 0.001, and ∗∗∗∗ for p < 0.0001.
