# Supplementary material for: Regulation of the DLC3 tumor suppressor by a novel phosphoswitch
Source: iScience. 2024 Jun 6;27(7):110203. doi: 10.1016/j.isci.2024.110203 (PMC11253157; doi:10.1016/j.isci.2024.110203)
Supplement: Document S1. Figures S1–S5 and Table S1 [file mmc1.pdf]

iScience, Volume *27*

Supplemental information

Regulation of the DLC3 tumor suppressor by a novel phosphoswitch

Yannick Frey, Cristiana Lungu, Florian Meyer, Franziskus Hauth, Daniel Hahn, Corinna Kersten, Vivien Heller, Mirita Franz-Wachtel, Boris Macek, Igor Barsukov, and Monilola A. Olayioye

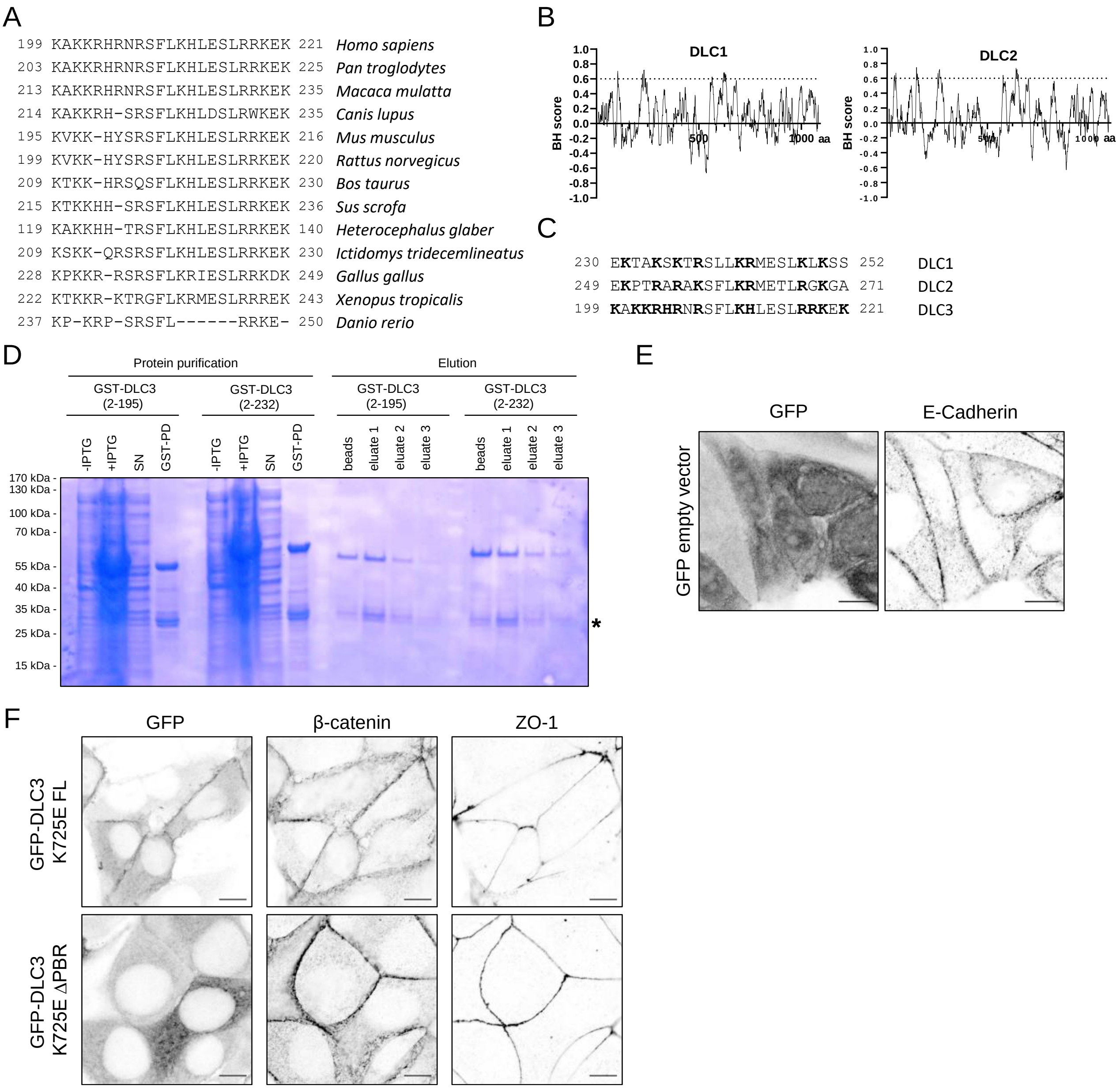

Supplemental Figure S1. Regulation of membrane association by the DLC3 PBR, Related to Figure 1.

(A) Sequence comparison of DLC3 orthologue PBR amino acid sequences from the indicated species. (B) BH plot of basic and hydrophobic residues in the DLC1 and DLC2 sequence using the scale developed by Brzeska et al. (C) Sequence comparison of the novel PBR in DLC3 with homologous regions of other DLC family members after alignment with BLAST. Basic amino acids are marked in bold. (D) Purification of recombinant GST-tagged N-terminal DLC3 fragments from *E. coli*. –IPTG: before induction; +IPTG: after induction; SN: supernatant after GST-pulldown; GST-PD: protein bound to beads after GST-pulldown; beads: protein remaining on beads; eluate 1-3: eluted protein fractions. Samples were analyzed by SDS-PAGE followed by Coomassie staining. Asterisk denotes free GST cleavage products. (E) Expression of GFP in stable MCF7 cells was induced for 24 h with doxycycline, cells were fixed and analyzed by immunofluorescence microscopy. E-cadherin immunostainings. Images are maximum intensity projections of several confocal sections. Scale bars: 10 µm. (F) Expression of indicated GFP-tagged DLC3 constructs in stable MCF7 cells was induced for 24 h with doxycycline, cells were fixed and analyzed by immunofluorescence microscopy. β-catenin- and ZO-1-

specific immunostainings. Images are maximum intensity projections of several confocal sections. Scale bars: 10 µm.

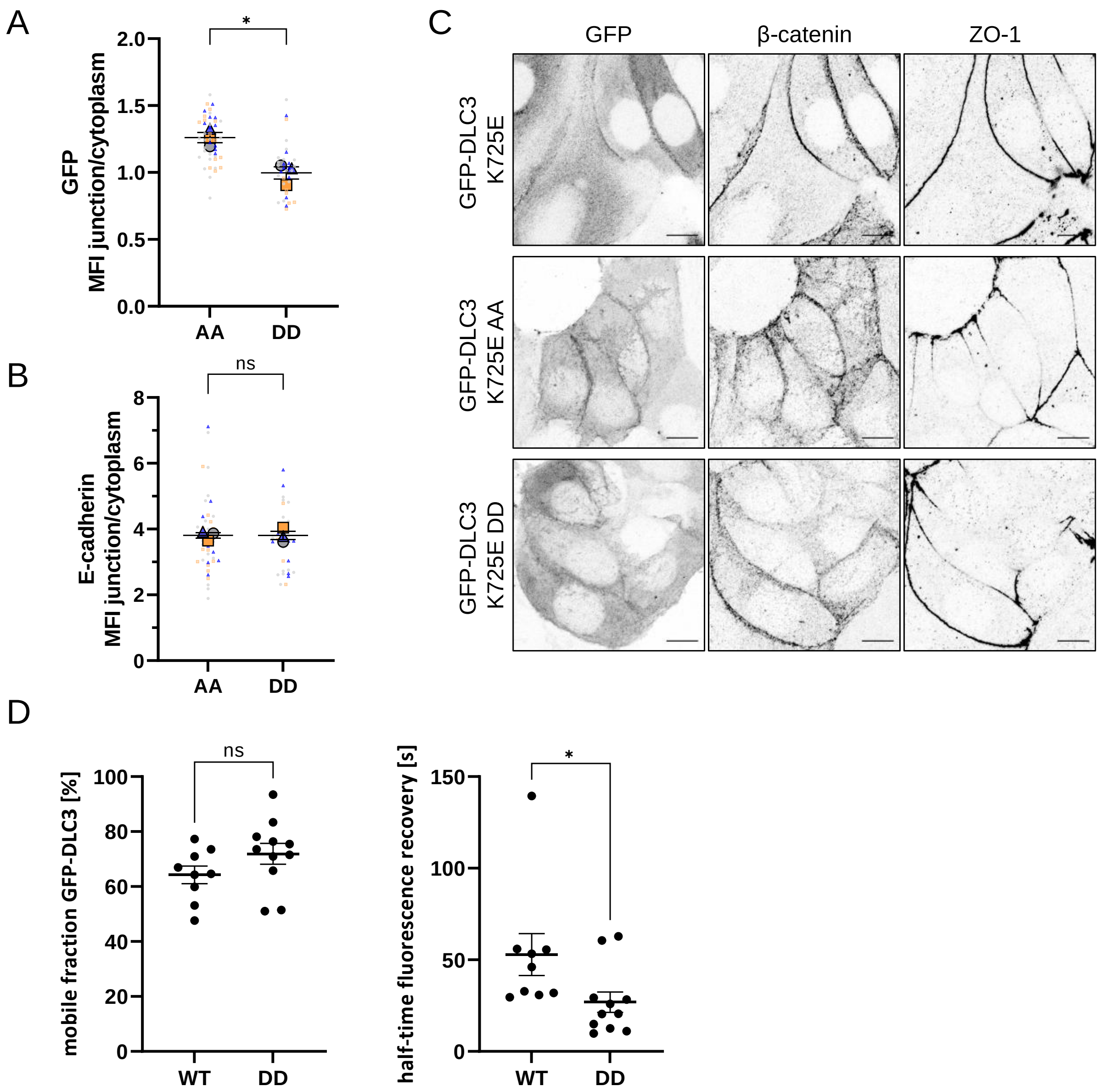

Supplemental Figure S2. Phospomimetic DLC3 PBR mutants show impaired membrane association *in cellulo*, Related to Figure 3.

(A/B) Localization GFP-DLC3 K725E phosphodeficient (AA) and phosphomimetic (DD) PBR mutants analyzed in MCF7 cells inducibly expressing the constructs with E-cadherin-specific immunostainings. Graph shows the mean fluorescence intensity (MFI ± SEM) of the signal at cell junctions versus the cytoplasmic signal for GFP (A) or E-cadherin (B) (n=3; N=43, 33 cells; t-test: p=0.0114 (A), p=0.9993, ns=not significant (B)). (C) Expression of indicated GFP-tagged DLC3 constructs in stable MCF7 cells was induced for 24 h with doxycycline, cells were fixed and analyzed by immunofluorescence microscopy. β-catenin- and ZO-1- specific immunostainings. Images are maximum intensity projections of several confocal sections. Scale bars: 10 µm. (D) Individual intensity curves from FRAP experiments in Figure 3D were analyzed by one-phase association nonlinear regression to obtain half-time of fluorescence recovery (t_{half}) and mobile fraction (plateau) (N= 9, 11; t-test t_{half}: p=0.0445; t-test plateau: p=0.1485, ns=not significant; values show mean ± SEM).

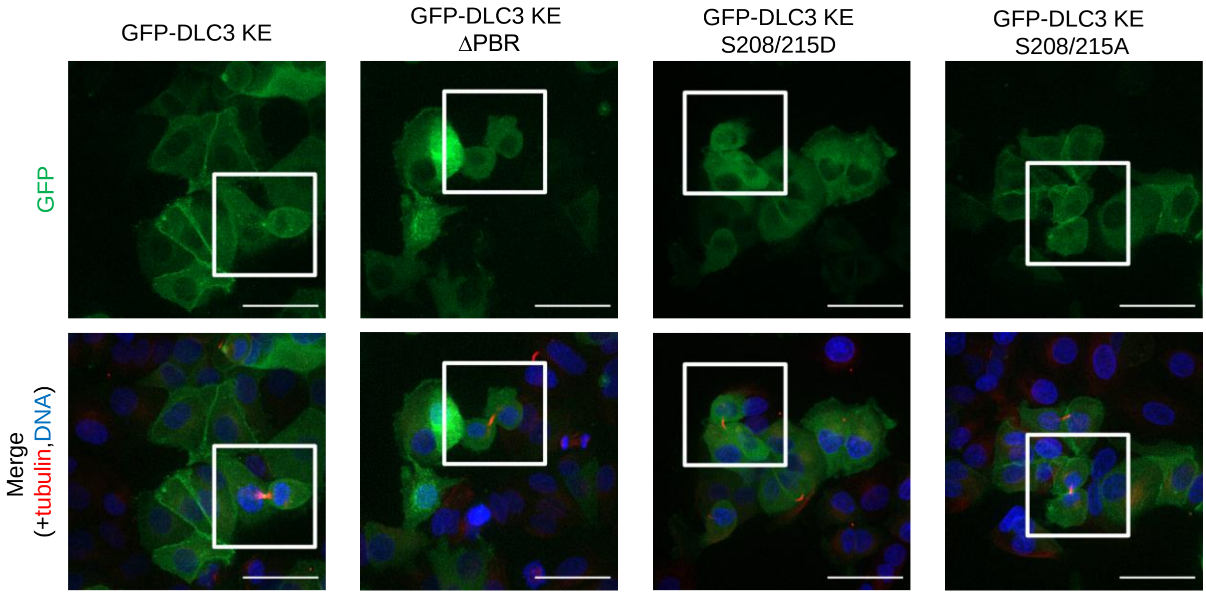

Supplemental Figure S3. Uncropped immunofluorescence microscopy images of dividing cells expressing different GFP-DLC3 constructs, Related to Figure 4B.

Expression of indicated GFP-tagged DLC3 constructs (green) in stable MCF7 cells was induced for 24 h with doxycycline and cells were analyzed by live-cell imaging. Midbodies were identified using SPY555-tubulin staining (red). Nuclei were counterstained with SPY650-DNA. Scale bars: 50 µm.

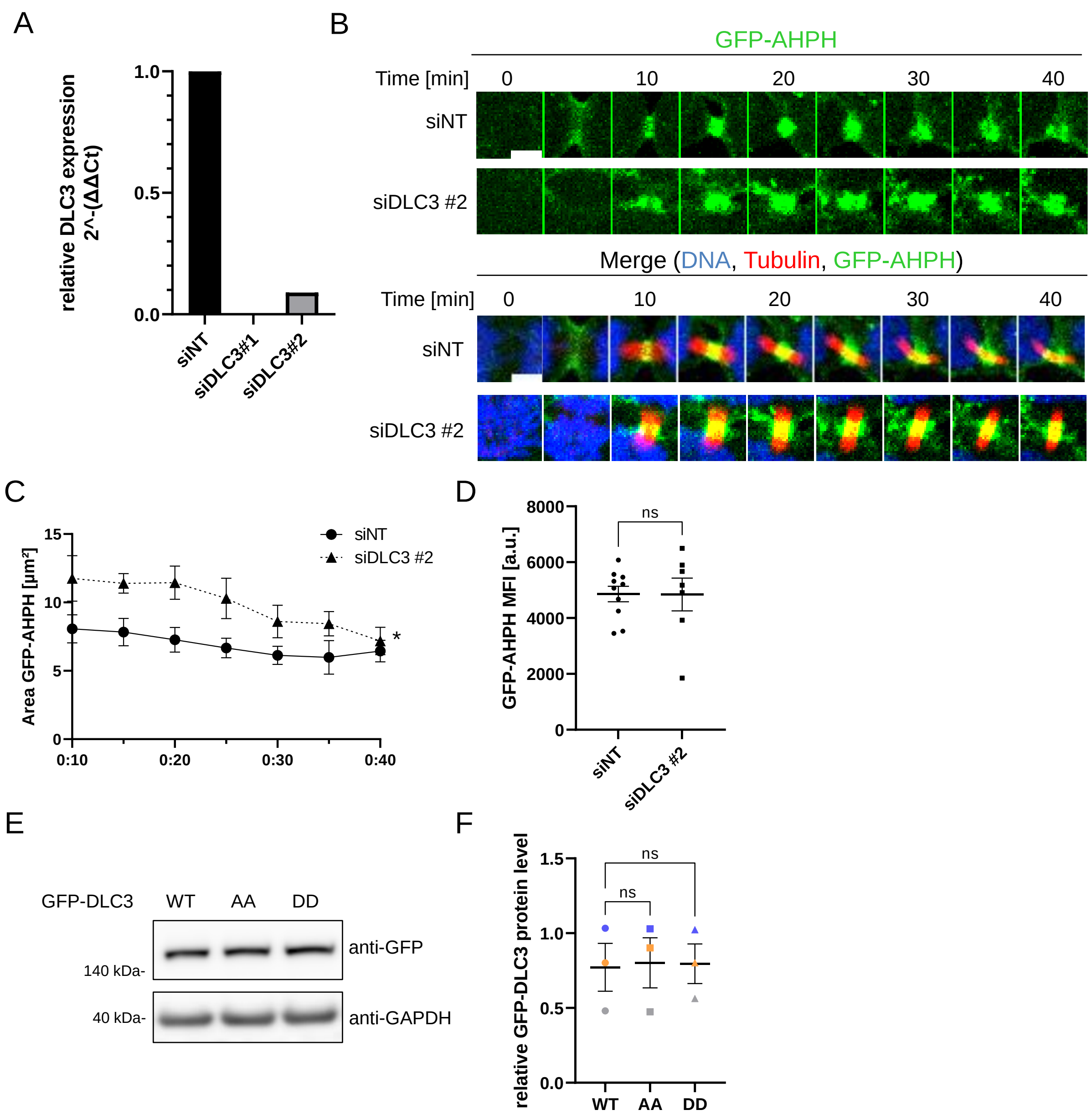

Supplemental Figure S4. Phosphomimetic DLC3 PBR mutants show impaired membrane association *in cellulo*, Related to Figure 4.

(A) qPCR analysis of DLC3 expression in MCF7 cells 72h after transfection with the indicated siRNAs. GAPDH was used as a reference gene, expression was normalized to non-targeting control siRNA. Shown is one representative experiment. (B) MCF7 cells stably expressing the Rho activity sensor GFP-AHPH (green) were transfected with the indicated siRNAs. After 72 h, fresh medium containing SPY555-tubulin (red) and SPY650-DNA (blue) was added and cells were analyzed by live-cell fluorescence microscopy. Scale bar = 5 µM. (C) Datapoints show the mean area of the GFP-AHPH signal at the midbody at a given timepoint [h:mm] (bars represent SEM; N=10, 6; Mixed-effects analysis of Two-way ANOVA with repeated measurements and Geisser-Greenhouse correction: p=0.025). (D) Mean fluorescence intensities of GFP-AHPH signal at individual midbodies during the time frame shown in (C) (N=10, 6; mean ± SEM; t-test: p=0,9817; ns=not significant). (E) Expression of indicated GFP-tagged DLC3 constructs in stable MCF7 cells was induced with doxycycline. Cells were lysed and lysates were analyzed by immunoblotting with the indicated antibodies. (F) Western blots from three independent experiments from (E) were analyzed with ImageJ and the GFP signal was normalized to the respective

GAPDH signal (right panel). Samples from the same experiment are shown in the same color. Lines show mean ± SEM. One-way ANOVA with Dunnett's post-test: WT vs. AA $p=0.9862$, WT vs. DD $p=0.9912$.

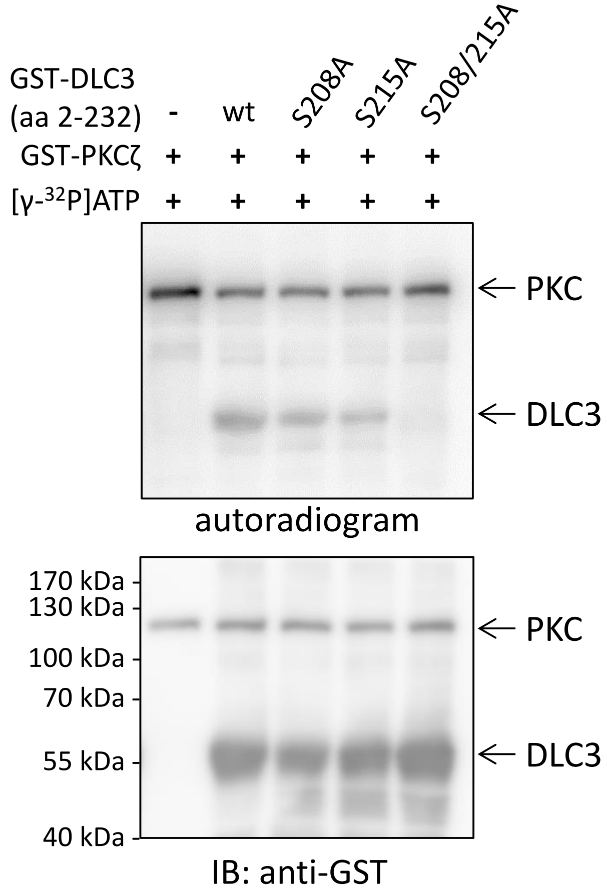

Supplemental Figure S5. aPKC phosphorylates serine residues in the DLC3 PBR *in vitro*, Related to Discussion.

In-vitro kinase assay on purified N-terminal DLC3-GST fusion proteins with or without phosphodeficient serine to alanine mutations using radiolabeled ATP and recombinant GST-PKCζ. Proteins were separated by SDS-PAGE and transferred to a PVDF membrane. Incorporation of radioactive phosphate was analyzed using a PhosphorImager (top panel), followed by immunoblotting with a GST-specific antibody (lower panel).

Supplemental Table S1. List of oligonucleotides used for cloning, site directed mutagenesis and quantitative real-time PCR, Related to STAR Methods.

Primer	Sequence 5' -> 3'
DLC3 aa2-232 fw	CCGGAATTCTCCTCTGCTGGACGTTTTCTG
DLC3 aa2-232 rv	CCGGAATTCTCAACTATGCTTGGGCTCTGCTTG
DLC3 aa2-195 fw	CCGGAATTCTCCTCTGCTGGACGTTTTCTG
DLC3 aa2-195 rv	CCGGAATTCTCAACCCTCCTGGCCCTGGGT
DLC3 ΔPBR fw	CAGGGCCAGGAGGGTCCAGCCACCTCAGAG
DLC3 ΔPBR rv	CTCTGAGGTGGCTGGACCCTCCTGGCCCTGT
DLC3 S208A fw	GCGCCATCGTAACCGTGCCTTCCTCAAGCACC
DLC3 S208A rv	GGTGCTTGAGGAAGGCACGGTTACGATGGCGC
DLC3 S215A fw	CAAGCACCTTGAAGCTCTGAGGCGGAAGG
DLC3 S215A rv	CCTTCCGCCTCAGAGCTTCAAGGTGCTTG
DLC3 S208D fw	GCGCCATCGTAACCGTGACTTCCTCAAGCACC
DLC3 S208D rv	GGTGCTTGAGGAAGTCACGGTTACGATGGCGC
DLC3 S215D fw	CTTCCTCAAGCACCTTGAAGATCTGAGGCGGAAGGAAAAG
DLC3 S215D rv	CTTTTCCTTCCGCCTCAGATCTTCAAGGTGCTTGAGGAAG
EGFP-AHPH fw	CCATTTCAGGTGTCCTGAGGATCATGGTGAGCAAGGGCGAG
EGFP-AHPH rv	CCGCCCTCGAGGAATTTCAAGGCTTTCCAATAGGTTTGTAGC
DLC3-F	CTGGACCAAGTAGGCATCTTCC
DLC3-R	CTCTTCCATGTAGAGGCTCAGG
GAPDH-F	CCCCTTCATTGACCTCAACTA
GAPDH-R	CGCTCCTGGAAGATGGTGAT
